# Development of diagnostic tools and discovery of two novel Indian citrus ringspot virus species: insights into global mandarivirus phylogeography

**DOI:** 10.3389/fmicb.2025.1513291

**Published:** 2025-02-14

**Authors:** Rushikesh D. Bharsakale, Mrugendra G. Gubyad, Krishanu Mukherjee, Amol D. Kokane, Sunil B. Kokane, Pragati Misra, Dilip Kumar Ghosh

**Affiliations:** ^1^Plant Virology Laboratory, ICAR-Central Citrus Research Institute, Nagpur, Maharashtra, India; ^2^Whitney Laboratory for Marine Biosciences, University of Florida, St. Augustine, FL, United States; ^3^Department of Molecular and Cellular Engineering, Sam Higginbottom University of Agriculture, Technology, and Sciences, Prayagraj, Uttar Pradesh, India

**Keywords:** Citrus yellow vein clearing virus, Indian citrus ringspot virus, RT-PCR diagnostics, phylogenetic diversity, emerging citrus pathogens

## Abstract

Emerging viral pathogens, Indian citrus ringspot virus (ICRSV) and Citrus yellow vein clearing virus (CYVCV), pose significant threats to global citrus production, a cornerstone of agriculture and trade. The rapid transboundary spread of CYVCV underscores the urgent need for effective diagnostic tools. To tackle this issue, we developed a novel RT-PCR assay capable of simultaneously detecting ICRSV and CYVCV with high sensitivity and specificity in a single reaction. This assay, targeting conserved genomic regions, provides a cost-effective, reliable, and scalable diagnostic solution. Field validation across 49 citrus samples revealed infection rates of 61.22% for ICRSV and 24.48% for CYVCV, with notable co-infections, highlighting its precision and utility. Phylogenetic analyses uncovered substantial genetic diversity, delineating two ICRSV species (ICRSV-A and ICRSV-B) and eight geographically clustered CYVCV clades, reflecting trade-driven and environmental dispersal patterns. These findings emphasize the need for region-specific diagnostics and tailored management strategies. By addressing critical diagnostic gaps, this assay enables early detection and intervention, reducing economic losses and enhancing efforts to control emerging citrus pathogens. Additionally, it provides a foundation for future epidemiological research and contributes to the sustainability of global citrus production.

## Introduction

Citrus crops, cultivated in tropical, subtropical, and temperate regions, are among the most economically significant fruit crops globally. Global production reached 158.5 million metric tons during the 2021/22 season, with China contributing 28%, followed by Brazil and India (FAO, 2021). These crops are vital to agricultural economies, producing widely consumed fruits such as oranges (*Citrus sinensis*), mandarins (*Citrus reticulata*), lemons (*Citrus limon*), and limes (*Citrus aurantiifolia*). However, citrus production faces mounting challenges from emerging viral pathogens that significantly threaten yield and quality. Among these, two critical threats are Citrus yellow vein clearing virus (CYVCV) and Indian citrus ringspot virus (ICRSV). These viruses cause severe yield losses and fruit quality deterioration, particularly in Asia, and their geographic expansion complicates management efforts (Ghosh et al., 2021; Kokane et al., 2020a; Prabha and Baranwal, 2011) The increasing prevalence of these pathogens underscores the urgent need for precise and reliable diagnostic tools to enable early detection and to support effective management strategies.

First identified in Pakistan in 1988, Citrus yellow vein clearing virus (CYVCV) has emerged as a major threat to citrus production, spreading extensively across Asia and other parts of the Middle East. It has reached key citrus-growing regions, including India, Türkiye, Iran, and China, with recent detections in South Korea and the state of California in the United States (Abrahamian et al., 2024; Jin et al., 2024; Sun and Yokomi, 2024; Zhou et al., 2017). CYVCV is now widespread in most citrus-producing areas of China, where research has focused on its molecular characteristics, transmission pathways, and management strategies (Liu et al., 2020; Zhou et al., 2017). Capable of infecting multiple citrus species, the virus causes substantial economic losses in affected regions. Its inclusion on the European and Mediterranean Plant Protection Organization (EPPO) Alert List after detection in Campania, Italy, in 2024 highlights its growing threat and raises concerns about its potential to disrupt citrus industries across the Mediterranean region (EPPO, 2024).

The distinct biological characteristics and regional impacts of CYVCV and ICRSV highlight the varied challenges they pose to the citrus industry. CYVCV manifests as yellow vein clearing, water-soaked veins, leaf distortion, and chlorosis, particularly in regions where susceptible cultivars are grown. These symptoms are especially severe in highly susceptible cultivars like Etrog citron, which is often used as an indicator host (Catara et al., 1993). In contrast, ICRSV, predominantly confined to India, causes distinct ringspot lesions and chlorotic patterns on leaves and fruits, leading to significant reductions in productivity and market value (Ahlawat and Pant, 2003; Prabha and Baranwal, 2012). While CYVCV’s rapid spread poses an international threat, ICRSV’s localized but impactful presence in India, raises concerns about its potential to disrupt regional citrus production, as it has not yet been reported outside the country (Kokane et al., 2020b; Rustici et al., 2000). Together, these emerging pathogens underscore the urgent need for innovative diagnostic tools and effective management strategies to mitigate their impacts and prevent further dissemination.

Both CYVCV and ICRSV belong to the genus Mandarivirus within the family Alphaflexiviridae. They are primarily transmitted through mechanical inoculation, grafting, and sap transmission, with aphid vectors also suspected to play a role in their spread. Unlike many other plant viruses, they are not seed-transmitted, which underscoring the importance of human-mediated activities and vector control in limiting their dissemination (Afloukou et al., 2021; Maghsoudi et al., 2023; Meena et al., 2020; Zhang Y. et al., 2019; Zhang Y. H. et al., 2019; Zhou et al., 2015). The global trade of citrus planting material and increased human activity have further exacerbated the spread of these viruses, complicating containment efforts (Warghane et al., 2020). However, despite their growing prevalence and impact, the evolutionary dynamics and transmission pathways of CYVCV remain poorly understood, highlighting the urgent need for targeted research to fill critical knowledge gaps.

The challenge of managing CYVCV and ICRSV is further intensified by co-infections with other citrus pathogens, such as Citrus tristeza virus (CTV) and Citrus yellow mosaic virus (CYMV). Co-infected plants often exhibit exacerbated symptoms, complicating effective disease management. These challenges underscore the need for diagnostic tools that are not only reliable and sensitive but also capable of detecting multiple pathogens simultaneously—a critical step toward enhanced citrus disease management strategies (Ghosh et al., 2020; Jin et al., 2024). While efforts to characterize the genetic diversity of CYVCV and ICRSV in specific regions have yielded valuable insights, comprehensive phylogenetic studies integrating global viral sequences remain limited. For instance, research on CYVCV has revealed significant genetic variability, with sequence identities ranging from 95.2 to 98.8% among variants from China, South Korea, and Pakistan (Jin et al., 2024). Similarly, although ICRSV demonstrates considerable genetic diversity within India, it remains uncertain whether these variations represent distinct species or intraspecific differences (Kokane et al., 2020b). This limited phylogenetic understanding hampers the development of precise diagnostic and management strategies, reinforcing the urgent need for further research into their evolutionary dynamics and transmission pathways.

Advancements in molecular diagnostics, such as multiplex PCR (mPCR) and SYBR Green-based RT-qPCR assays, have become powerful tools for detecting multiple viral pathogens simultaneously (Kokane et al., 2021; Meena and Baranwal, 2016). Classical methods like biological indexing and ELISA are reliable but limited by time-consuming protocol, reliance on indicator hosts, and reduced specificity for closely related strains (Meena and Baranwal, 2016; Steel et al., 2010). RPA-based diagnostics show promise but face high reagent costs and lack local production in India (Ghosh et al., 2020), while RT-qPCR remains sensitive but labor-intensive and expensive. Multiplex PCR (mPCR) addresses these challenges by offering cost-effective and robust detection of multiple pathogens in a single reaction tube. Successful mPCR assays have identified citrus viroids, viruses, and the HLB bacterium (Baranwal et al., 2005; Ito et al., 2002). This study advances diagnostics by targeting conserved regions within the coat protein (CP) and RNA-dependent RNA polymerase (RdRp) genes, enabling simultaneous detection of CYVCV and ICRSV to address critical challenges in citrus pathogen management.

In addition to advancing diagnostic tools, this study’s phylogenetic analyses of the CP and RdRp regions provide valuable insights into the evolutionary dynamics of CYVCV and ICRSV. Distinct CYVCV clades were identified, linked to geographical regions across Asia, the Middle East, and North America, while ICRSV showed significant sequence divergence marked by key insertions and deletions in the CP gene, revealing two genetically distinct species—ICRSV-A and ICRSV-B. These findings not only enrich our understanding of the genetic variability and spread of these pathogens but also enhance diagnostic precision, supporting more effective and targeted management strategies for citrus diseases globally.

## Results

### Design of novel primers for the simultaneous detection of mandarivirus: targeting the coat protein genes of both ICRSV and CYVCV

**Figure 1:**
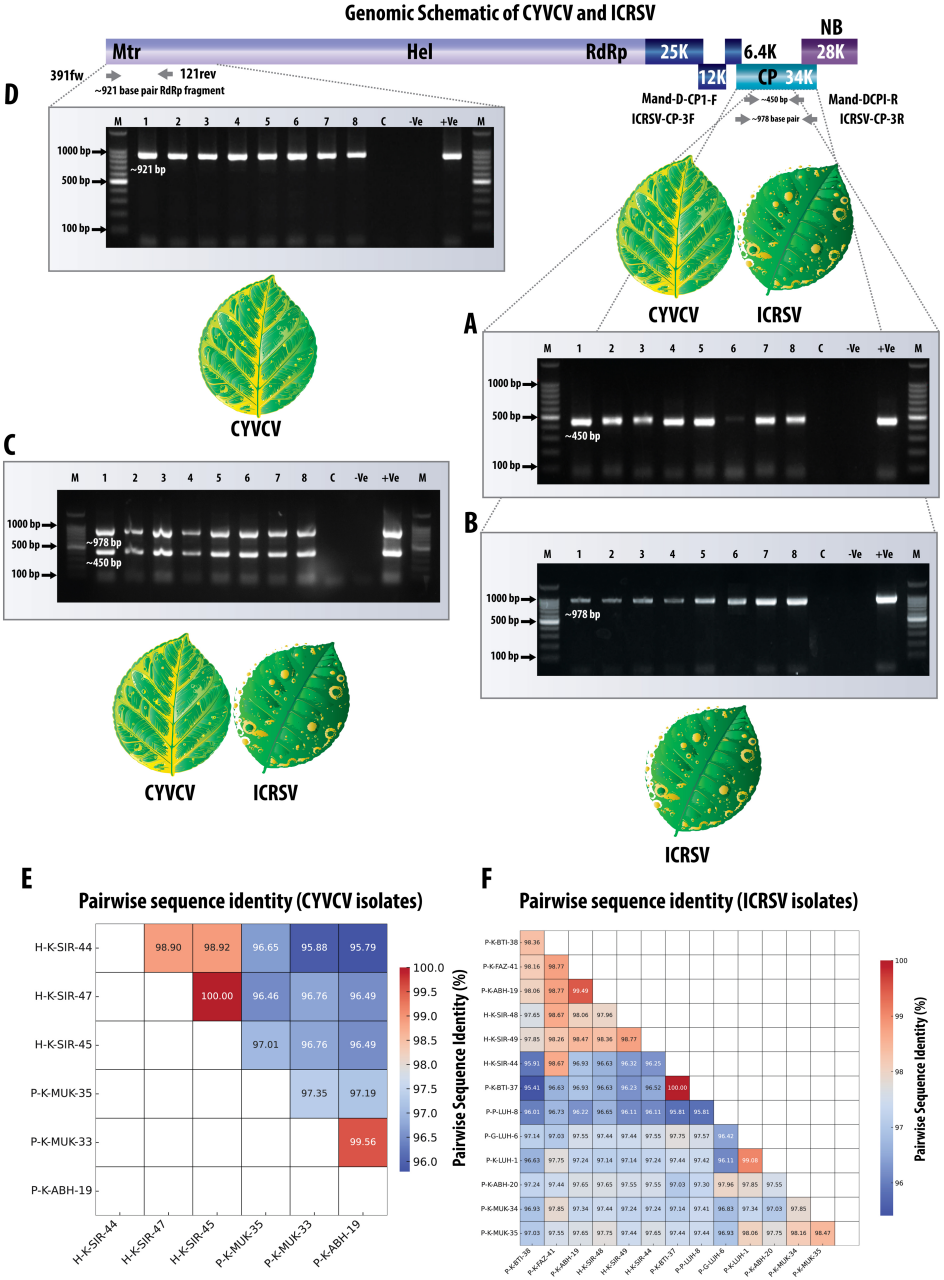
Comprehensive detection and validation of CYVCV and ICRSV Using RT-PCR assays. The top panel shows a schematic representation of the viral genome, highlighting the targeted regions for RT-PCR amplification. Due to the high sequence similarity and conserved domain architecture between CYVCV and ICRSV, a single genome representation is used, illustrating shared coat protein (CP) and RNA-dependent RNA polymerase (RdRp) regions. **(A)** Gel electrophoresis image showing the RT-PCR amplified product (~450 bp) targeting the coat protein (CP) gene of Mandarivirus (CYVCV and ICRSV) using the dual-target primers Mand-D-CP1-F and Mand-D-CP1-R. *lane* M contains a 100 bp DNA ladder; lanes 1–8 display amplified products from representative variants. Lane C is the healthy plant control, -Ve is the negative control (no template), and + Ve is the positive control. **(B)** Gel electrophoresis image of the RT-PCR amplified product (~978 bp) targeting the ICRSV coat protein (CP) gene using ICRSV-specific primers (ICRSV-CP-3F/3R). The figure demonstrates successful detection of ICRSV in relevant samples. Lanes 1–8 correspond to the same samples as in **(A)**. **(C)** Gel electrophoresis image showing detection of CYVCV (~450 bp band) and ICRSV (~978 bp band) in a single PCR reaction using dual-target primers and ICRSV-specific primers. Dual bands indicate the presence of both viruses. Negative controls, including healthy plant samples and the NTC, show no amplification. Although samples containing only CYVCV would produce a single ~450 bp band, this scenario is not represented in the gel image. **(D)** Gel electrophoresis image showing the RT-PCR amplified product (~921 bp) targeting the RNA-dependent RNA polymerase (RdRp) gene of CYVCV using CYVCV-specific primers (391fw/121rev). Lanes 1–8 represent representative samples; the absence of bands in negative controls confirms primer specificity. **(E)** Heatmap illustrating pairwise sequence identity of six CYVCV RdRp variants amplified and sequenced in this study, with identity values ranging from 95 to 100%. The CYVCV RdRp gene sequences and their corresponding GenBank accession numbers are as follows: P-K-ABH-19: MW380946; P-K-MUK-33: MW380943; P-K-MUK-35: MW380941; H-K-SIR-45: MW380945; H-K-SIR-44: MW380942; H-K-SIR-47: MW380944. **(F)** Heatmap illustrating pairwise sequence identity of 13 ICRSV CP variants amplified and sequenced in this study, with identity values ranging from 95 to 100%. The ICRSV CP gene sequences and their corresponding GenBank accession numbers are as follows: P-K-BTI-38: MW233695; P-K-FAZ-41: MW233701; P-K-ABH-19: MW233704; H-K-SIR-48: MW233698; H-K-SIR-49: MW233700; H-K-SIR-44: MW233691; P-K-BTI-37: MW233692; P–P-LUH-8: MW233694; P-G-LUH-6: MW233693; P-K-LUH-1: MW233703; P-K-ABH-20: MW233702; P-K-MUK-34: MW233696; P-K-MUK-35: MW233697. This comprehensive validation highlights the reliability and accuracy of the primers in detecting both viruses across diverse field samples.

A novel primer pair targeting the coat protein (CP) region was developed to simultaneously detect both Indian citrus ringspot virus (ICRSV) and Citrus yellow vein clearing virus (CYVCV). These primers, referred to as dual-target primers, amplify a conserved ~450 bp fragment, enabling the detection of both viruses in a single RT-PCR reaction. The primer design was informed by a multiple sequence alignment (MSA) of CP gene sequences from ICRSV and CYVCV, ensuring specificity for both viruses while avoiding cross-reactivity with unrelated citrus pathogens. The sequences of the primers are provided in Table 1. Extensive testing was conducted to validate the sensitivity and specificity of the primers across controlled and field samples. Initial validation in positive control samples from a screen house demonstrated consistent amplification of the target region, confirming the high specificity of the primers for both viruses. This robustness was further demonstrated by field validation across 49 citrus samples, where 34 samples produced the expected ~450 bp amplicons (Figure 1A). The absence of amplification in healthy plant controls and non-template controls demonstrated the assay’s high specificity, minimizing the occurrence of false positives. The RT-PCR assay utilizing this novel primer set effectively detected both ICRSV and CYVCV in single and mixed-virus infections (Table 2). In specificity testing, the primers were assessed against a broad range of citrus pathogens, including Citrus tristeza virus (CTV), Citrus yellow mosaic virus (CYMV), *Candidatus* Liberibacter asiaticus (*C*Las), and citrus phytoplasma. No amplification was observed in RT-PCR reactions involving these pathogens or in healthy control samples, underscoring the high selectivity of the primers for ICRSV and CYVCV. Sequencing of RT-PCR products confirmed the presence of both ICRSV and CYVCV in all samples where amplification occurred, ensuring the accuracy of virus detection. These findings highlight the precision, reliability, and practical utility of the novel primers for the simultaneous detection of these two citrus pathogens, both in controlled laboratory conditions and diverse field settings.

**Table 1:** Nucleotide sequences, target gene, and expected amplicon sizes of primer pairs used in RT-PCR assays.

Sr no	Primer name	Primer sequence (5′–3′)	Length	Tm (°C)	GC (%)	Amplicon size (nt)	Targeted region
1	Mand-D-CP1-F	CTAACGACACGACCCCGAAA	20	54	55.0	⁓451 bp	Coat protein
	Mand-D-CP1-R	AGCGGGCAGAAATCCCTAAC	20	54	55.0		
2	ICRSV-CP-3F	CTCATGAGCTTTGACTACAC	20	50	45.0	⁓978 bp	Coat protein
	ICRSV-CP-3R	CACACGCACACGATATTATGGTAA	24	54	41.67		
3	391fw	GAAAAGCAAACAGTAACAAACACACCC	27	57	40.74	⁓921 bp	RdRp
	121rev	GGGCAAGAGCATTTGGGTATCT	22	55	50.0		

**Table 2:** Validation of novel primers using field-collected along with biologically indexed citrus samples, and their RT-PCR results using the Mand-D-CP1-F/R (novel primer), ICRSV-CP-3F/3R (ICRSV), 391fw/121rev (CYVCV).

Sr no	Sample ID	Citrus cultivar	Location	RT-PCR detection		
				Mandariviruses	ICRSV	CYVCV
1.	Abo-1	*C. reticulata*	Abohar	−	−	−
2.	Abo-2	*C. reticulata*	Abohar	+	−	+
3.	Abo-3	*C. reticulata*	Abohar	−	−	−
4.	Abo-4	*C. reticulata*	Abohar	+	+	+
5.	Abo-5	*C. sinensis*	Abohar	+	+	−
6.	Abo-6	*C. sinensis*	Abohar	−	−	−
7.	Abo-7	*C. reticulata*	Abohar	+	+	−
8.	Abo-8	*C. reticulata*	Abohar	−	−	−
9.	Abo-9	*C. reticulata*	Abohar	+	+	+
10.	Abo-10	*C. reticulata*	Abohar	+	+	−
11.	Abo-11	*C. reticulata*	Abohar	+	+	−
12.	Abo-12	*C. reticulata*	Abohar	−	−	−
13.	Abo-13	*C. reticulata*	Abohar	+	+	−
14.	Abo-14	*C. reticulata*	Abohar	+	+	−
15.	Abo-15	*C. reticulata*	Abohar	+	+	−
16.	Bat-16	*C. reticulata*	Bathinda	+	+	−
17.	Bat-17	*C. reticulata*	Bathinda	+	+	−
18.	Bat-18	*C. reticulata*	Bathinda	+	+	−
19.	Bat-19	*C. reticulata*	Bathinda	−	−	−
20.	Sir-20	*C. reticulata*	Sirsa	+	+	+
21.	Sir-21	*C. reticulata*	Sirsa	+	−	+
22.	Sir-22	*C. reticulata*	Sirsa	−	−	−
23.	Sir-23	*C. reticulata*	Sirsa	+	+	+
24.	Sir-24	*C. reticulata*	Sirsa	+	+	+
25.	Sir-25	*C. reticulata*	Sirsa	+	+	−
26.	Hos-26	*C. reticulata*	Hoshiarpur	+	+	−
27.	Hos-27	*C. reticulata*	Hoshiarpur	−	−	−
28.	Hos-28	*C. reticulata*	Hoshiarpur	−	−	−
29.	Hos-29	*C. reticulata*	Hoshiarpur	+	+	−
30.	Hos-30	*C. reticulata*	Hoshiarpur	+	+	−
31.	Lud-31	*C. reticulata*	Ludhiana	+	+	−
32.	Lud-32	*C. reticulata*	Ludhiana	−	−	−
33.	Lud-33	*C. reticulata*	Ludhiana	+	+	+
34.	Lud-34	*C. reticulata*	Ludhiana	+	+	−
35.	Lud-35	*C. reticulata*	Ludhiana	−	−	−
36.	Lud-36	*C. paradisi*	Ludhiana	+	+	−
37.	Lud-37	*C. paradisi*	Ludhiana	−	−	−
38.	Lud-38	*C. grandis*	Ludhiana	+	−	+
39.	Lud-39	*C. reticulata*	Ludhiana	+	+	−
40.	Lud-40	*C. reticulata*	Ludhiana	−	−	−
41.	Faz-41	*C. reticulata*	Fazilka	+	+	−
42.	Faz-42	*C. reticulata*	Fazilka	+	+	−
43.	Faz-43	*C. reticulata*	Fazilka	+	−	+
44.	Muk-44	*C. reticulata*	Muktsar	−	−	−
45.	Muk-45	*C. reticulata*	Muktsar	+	+	−
46.	Muk-46	*C. reticulata*	Muktsar	+	−	+
47.	Muk-47	*C. reticulata*	Muktsar	+	+	−
48.	Muk-48	*C. reticulata*	Muktsar	+	+	+
49.	Muk-49	*C. reticulata*	Muktsar	−	−	−
50.	Nag-50 (Healthy)	*C. sinensis*	Nagpur	−	−	−
Positive control						
51.	ICRSV-Nag-1	*C. reticulata*	Nagpur	+	+	−
52.	ICRSV-Nag-2	*C. reticulata*	Nagpur	+	−	−
53.	ICRSV-Nag-3	*C. reticulata*	Nagpur	+	+	−
54.	ICRSV-Nag-4	*C. reticulata*	Nagpur	+	+	−
55.	ICRSV-Nag-5	*C. sinensis*	Nagpur	+	+	−
56.	ICRSV-Nag-6	*C. sinensis*	Nagpur	+	+	−
57.	ICRSV-Nag-7	*C. sinensis*	Nagpur	+	+	−
58.	CYVCV-Nag-1	*C. reticulata*	Nagpur	+	−	+
59.	CYVCV-Nag-2	*C. reticulata*	Nagpur	+	−	+
60.	CYVCV-Nag-3	*C. reticulata*	Nagpur	−	−	+
61.	CYVCV-Nag-4	*C. reticulata*	Nagpur	+	−	+
Other major citrus pathogens						
62.	CTV-1	*C. aurantifolia*	Nagpur	−	−	−
63.	CTV-2	*C. sinensis*	Nagpur	−	−	−
64.	CYMV-1	*C. sinensis*	Nagpur	−	−	−
65.	CYMV-2	*C. sinensis*	Nagpur	−	−	−
66.	*C*Las-1	*C. sinensis*	Nagpur	−	−	−
67.	*C*Las-2	*C. sinensis*	Nagpur	−	−	−
68.	Phytoplasma-1	*C. aurantifolia*	Nagpur	−	−	−
69.	Phytoplasma-2	*C. aurantifolia*	Nagpur	−	−	−

ICRSV, Indian citrus ringspot virus; CYVCV, Citrus yellow vein clearing virus; RT-PCR, Reverse transcription polymerase chain reaction; CTV, Citrus tristeza virus; CYMV, Citrus yellow mosaic virus; *C*Las, *Candidatus* Liberibacter asiaticus; (+) Positive; (−) Negative.

### Precise detection of Indian citrus ringspot virus (ICRSV) using a targeted CP gene-specific RT-PCR assay

To achieve accurate detection of Indian citrus ringspot virus (ICRSV), the 49 citrus samples previously analyzed with the dual-target primers were subjected to a reverse transcription-polymerase chain reaction (RT-PCR) assay developed in this study. The ICRSV-specific primers, ICRSV-CP-3F and ICRSV-CP-3R, were designed based on a multiple sequence alignment (MSA) of the coat protein (CP) regions of ICRSV and Citrus yellow vein clearing virus (CYVCV), ensuring they specifically amplify a region unique to ICRSV without cross-reactivity with CYVCV. These primers successfully amplified a ~ 978 bp fragment in 29 of the 49 samples, confirming the presence of ICRSV (Figure 1B). No amplification was observed in the remaining 20 samples, including 19 field samples that tested negative, the healthy citrus plant samples used as negative controls, and the non-template control (NTC). These results underscore the specificity and reliability of the ICRSV-specific primers in distinguishing ICRSV from other pathogens. To further validate the accuracy of the assay, 14 representative amplicons were selected for sequencing. The amplified products were carefully excised from the gel, purified, and sequenced. Comparative analysis of these sequences against viral genome data in the GenBank database using BLAST revealed high identity with previously reported ICRSV variants. All newly sequenced variants have been deposited into the GenBank database for future reference and accessibility (Table 2). These findings highlight the robustness and specificity of the RT-PCR assay developed in this study, demonstrating its practical utility for the precise detection of ICRSV in both controlled laboratory conditions and diverse field settings.

### Simultaneous detection of CYVCV and ICRSV using dual-target and ICRSV-specific primers in a single PCR reaction

To validate the simultaneous detection of Citrus yellow vein clearing virus (CYVCV) and Indian citrus ringspot virus (ICRSV), a single PCR reaction was performed for each of the previously tested 49 citrus samples. The reaction included both the dual-target primers (Mand-D-CP1-F and Mand-D-CP1-R) and the ICRSV-specific primers (ICRSV-CP-3F and ICRSV-CP-3R). This setup allowed for amplification of two distinct regions: the conserved coat protein (CP) region shared by both viruses (~450 bp) and the ICRSV-specific region (~978 bp). In samples containing both ICRSV and CYVCV, two distinct bands were observed: the ~450 bp band amplified by the dual-target primers and the ~978 bp band amplified by the ICRSV-specific primers (Figure 1C). In contrast, if a sample contained only CYVCV and no ICRSV, we would expect to see a single band at ~450 bp due to amplification of the conserved CP region. The absence of the ~978 bp band in such cases would confirm the absence of ICRSV. However, this scenario is not shown in the current gel image. Negative controls, including healthy citrus plant samples and the non-template control (NTC), showed no amplification, confirming the specificity of both primer sets. These results highlight the robustness of this dual-primer approach for detecting ICRSV and CYVCV simultaneously in mixed infections or distinguishing single-virus infections.

### Efficient detection of Citrus yellow vein clearing virus (CYVCV) using RNA-dependent RNA polymerase (RdRp) gene-specific primers

To achieve accurate detection of Citrus yellow vein clearing virus (CYVCV), the same set of 49 citrus samples was subjected to RT-PCR analysis using primers specifically designed to target the RNA-dependent RNA polymerase (RdRp) gene. These primers (391fw and 121rev) were developed based on multiple sequence alignment (MSA) of RdRp sequences from CYVCV and ICRSV. Conserved regions unique to CYVCV were identified to ensure that the primers specifically amplified CYVCV, without cross-reactivity with ICRSV. The assay successfully amplified the expected ~921 bp fragment in 12 samples, confirming the presence of CYVCV in these cases (Figure 1D). No amplification was observed in the remaining 37 samples, confirming the absence of CYVCV. Additionally, no amplification was detected in the healthy citrus plant samples used as negative controls or in the non-template control (NTC), demonstrating the high specificity of the RdRp gene-specific primers and eliminating the possibility of false positives. To further validate the accuracy of the RT-PCR results, six representative CYVCV-positive amplicons were excised from the gel, purified, and sequenced. Sequence analysis revealed high identity with previously reported CYVCV variants in GenBank, confirming the reliability of the detection method. These newly sequenced variants have been deposited into the GenBank database to expand the repository of CYVCV genomic data (Table 3), supporting future studies on the virus’s genetic diversity and epidemiology.

**Table 3:** GenBank accession numbers for Indian citrus ringspot virus (ICRSV) and Citrus yellow vein clearing virus (CYVCV) variants from India.

Sr. no.	Sample code	ICRSV accession number of CP gene	CYVCV ACCESSION number of RdRp gene
1.	P-K-LUH-1	MW233703	-
2.	P-G-LUH-6	MW233693	-
3.	P–P-LUH-8	MW233694	-
4.	P-K-ABH-19	MW233704	MW380946
5.	P-K-ABH-20	MW233702	-
6.	P-K-MUK-33	-	MW380943
7.	P-K-MUK-34	MW233696	-
8.	P-K-MUK-35	MW233697	MW380941
9.	P-K-BTI-37	MW233692	-
10.	P-K-BTI-38	MW233695	-
11.	P-K-FAZ-41	MW233701	-
12.	H-K-SIR-45	-	MW380945
13.	H-K-SIR-44	MW233691	MW380942
14.	H-K-SIR-47	MW233699	MW380944
15.	H-K-SIR-48	MW233698	SND
16.	H-K-SIR-49	MW233700	-

(−) indicates a negative sample; SND means sequencing was not done; CP refers to the coat protein gene; RdRp stands for RNA-dependent RNA polymerase gene.

### High sequence conservation in CYVCV and ICRSV variants across cDNA and protein levels revealed by pairwise identity analysis

**Figure 2:**
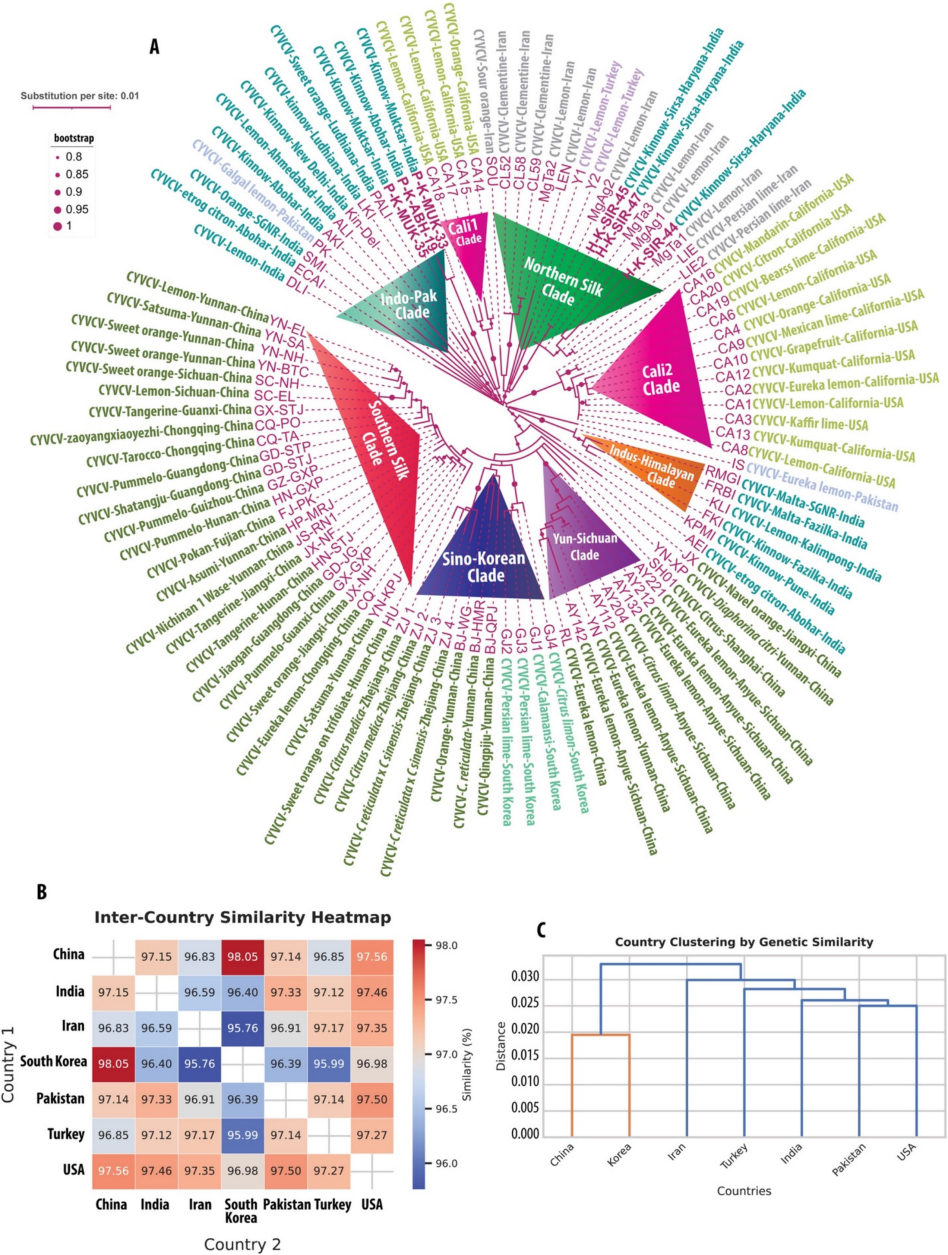
Geographic and genetic insights into Citrus yellow vein clearing virus (CYVCV). **(A)** Phylogenetic tree of CYVCV variants, constructed using Maximum Likelihood (ML) analysis of concatenated cDNA sequences comprising 280 amino acids from RNA-dependent RNA polymerase (RdRp) and 325 amino acids from the coat protein (CP). Sequences include both full-length and partial entries from NCBI GenBank (as of September 2024) and six new variants sequenced in this study, highlighted in bold. Variant names in purple follow a standardized naming system indicating virus, host, region, and origin. Bootstrap values >80% signify strongly supported clades, reflecting evolutionary lineages and geographic dispersal routes. **(B)** Heatmap displaying inter-country genetic similarity of CYVCV based on CP cDNA sequences. Pairwise similarities between all sequences from each country were averaged to compute inter-country similarities, capturing the relative genetic relatedness between regions. **(C)** Hierarchical clustering dendrogram of CYVCV genetic similarity between countries, derived from pairwise inter-country similarity scores. Countries with higher genetic similarity cluster closely, illustrating patterns of geographic spread and evolutionary connections. Distances are represented as substitutions per site, with branch lengths proportional to genetic differences. This integrated analysis highlights global CYVCV dispersal pathways and evolutionary relationships, emphasizing the virus’s geographic adaptation and spread across regions.

The pairwise sequence identity analysis of CYVCV and ICRSV variants revealed high levels of sequence conservation, particularly at the protein level. Among the six CYVCV variants amplified and sequenced in this study, sequence identity values ranged from 95 to 100%, indicating limited divergence in the protein-coding regions of experimental samples. This high conservation is mirrored at the cDNA level for the coat protein (CP) gene, reinforcing the stability of these regions. This conservation is visually represented as a heatmap (Figure 1E), with gradient color scales highlighting subtle differences between the variants. Similarly, for the 13 ICRSV variants analyzed, sequence identities also ranged from 95 to 100%, reflecting significant conservation in the protein-coding regions. The ICRSV heatmap (Figure 1F) underscores the close genetic relationships within the ICRSV population. Expanding on these findings, Figures 2B,C highlight the coat protein (CP) cDNA sequence-based genetic similarities and geographic clustering of CYVCV isolates. Figure 2B shows the inter-country similarity heatmap, revealing high intra-country conservation, particularly in regions like India, Pakistan, Iran, and Turkey, where sequence similarities exceeded 98%. Notable geographical trends were observed, with isolates from distant regions such as China, South Korea, and the USA exhibiting slightly lower similarities (97–98%). Figure 2C showcases hierarchical clustering, further confirming the genetic relatedness among neighboring countries and supporting distinct geographical clades, consistent with the virus’s dispersal patterns. Supplementary Figures 2, 3 further detail sequence similarities across both the CP and RNA-dependent RNA polymerase (RdRp) regions. The high sequence conservation observed in both regions reflects strong evolutionary constraints acting on CYVCV’s protein-coding genes. The RdRp region exhibited a comparable pattern of high conservation, with neighboring countries like India, Pakistan, and Iran displaying sequence similarities exceeding 97%. In contrast, isolates from geographically distant regions, including the USA, South Korea, and China, showed marginally lower conservation, reflecting gradual divergence influenced by geographic and evolutionary factors. Together, these findings corroborate the phylogenetic insights presented in Figure 2A, where the geographical spread of CYVCV is aligned with patterns of genetic similarity. This highlights the virus’s dissemination from India along two major routes: westward to Iran, Turkey, and the USA, and eastward to China and South Korea. These findings emphasize the conserved nature of CYVCV genes, with limited evolutionary divergence observed in both CP and RdRp regions. This high conservation suggests genetic stability, potentially contributing to the virus’s persistence, adaptability, and pathogenicity in citrus crops. These findings shed light on CYVCV’s global transmission patterns, evolutionary trends, and their significance for devising effective management strategies in citrus cultivation.

### Classification of two novel Indian citrus ringspot virus species: ICRSV-A and ICRSV-B

**Figure 3:**
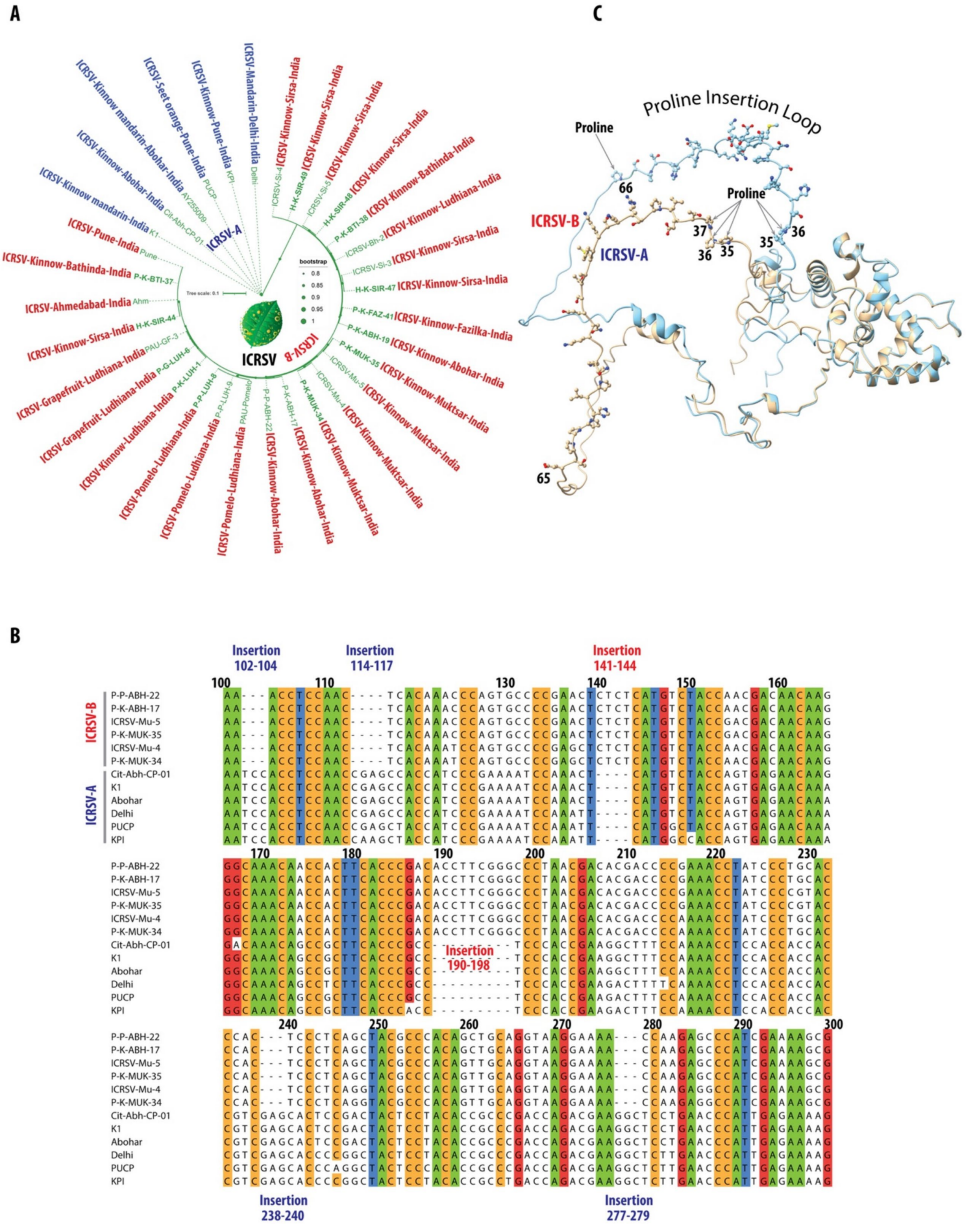
Phylogenetic and structural characterization of ICRSV-A and ICRSV-B: insights into evolutionary divergence and functional implications. **(A)** Maximum Likelihood (ML) phylogenetic tree of ICRSV coat protein gene sequences, created from DNA sequences due to their greater variability, revealing two distinct evolutionary lineages: ICRSV-A (blue) and ICRSV-B (red). The tree is supported by bootstrap values of 80% and higher, displayed at relevant nodes. These findings support the divergence of ICRSV-A and ICRSV-B as two separate species. The ICRSV-A sequences and their corresponding GenBank accession numbers are as follows: P-K-BTI-38: MW233695, P-K-FAZ-41: MW233701, P-K-ABH-19: MW233704, H-K-SIR-48: MW233698, H-K-SIR-49: MW233700, H-K-SIR-44: MW233691. Similarly, the ICRSV-B sequences and their corresponding GenBank accession numbers are as follows: ICRSV-Si-4: MN518766, ICRSV-Si-5: MN518764, ICRSV-Bh-2: MN518761, ICRSV-Si-3: MN518765, ICRSV-Mu-5: MN518763, ICRSV-Mu-4: MN518762, P-K-ABH-17: MW233702, P–P-ABH-22: MZ803193, PAU-Pomelo: MN518760, P–P-LUH-9: MZ803192, PAU-GF-3: MN422619, Ahm: AY255007, P-K-BTI-37: MW233692, P–P-LUH-8: MW233694, P-G-LUH-6: MW233693, P-K-LUH-1: MW233703, P-K-MUK-34: MW233696, P-K-MUK-35: MW233697, PUCP: HQ324250, K1: NC_003093. **(B)** DNA sequence alignment of the coat protein genes for representative variants from both ICRSV-A and ICRSV-B, focusing on the nucleotide positions between 100 and 300. The alignment is color-coded based on a sequence identity threshold of 90% and above. ICRSV-A exhibits several unique insertions at positions 102–104, 114–117, 238–240, and 277–279, while ICRSV-B features notable insertions at positions 141–144 and 190–198. These insertions may correspond to structural differences influencing the coat protein’s conformation and function. Insertions in ICRSV-A are highlighted in dark blue, and those in ICRSV-B are shown in red. **(C)** AlphaFold2-predicted structures of the coat proteins for ICRSV-A and ICRSV-B. The ICRSV-A coat protein is depicted in gold, while the ICRSV-B coat protein is shown in sky blue. The Proline Insertion Loop, spanning residues 35 to 66, is illustrated with all residues represented in ball-and-stick format. Proline residues at positions 35, 36, and 37 in ICRSV-A, as well as at positions 35 and 36 in ICRSV-B, are highlighted. Additionally, a proline insertion at position 66 in ICRSV-B is indicated with an arrow. This visualization underscores the structural differences between the two variants and their potential implications for the functionality of the coat proteins.

In this study, we conducted a comprehensive phylogenetic analysis of the coat protein gene using all GenBank-deposited ICRSV sequences, along with 13 variants that we sequenced (Table 3; Supplementary_Excel_Sheet_1.xlsx). This analysis revealed two genetically distinct clades, designated as ICRSV-A and ICRSV-B (Figure 3A). These clades exhibit clear divergence in sequence composition, supporting their classification as two novel ICRSV species. The Maximum Likelihood (ML) phylogenetic tree, constructed using the coat protein gene sequences, distinctly separates the variants into two groups with strong bootstrap support (>80%), confirming their genetic differentiation (Figure 3A). ICRSV-A contains six variants, all demonstrating high sequence identity within the group (>97%), while ICRSV-B consists of 26 variants with slightly lower sequence identity (>96%), indicating broader genetic diversity within this clade. The sequence identity between ICRSV-A and ICRSV-B is notably lower, ranging from 89 to 92%, further highlighting the genetic divergence between the two groups. The observed phylogenetic structure and bootstrap values strongly support the hypothesis that ICRSV-A and ICRSV-B represent distinct evolutionary lineages.

Further evidence for species-level classification arises from the analysis of insertions and deletions (indels) within the coat protein gene. The DNA sequence alignment of representative variants from both groups (Figure 3B) highlights multiple regions of variation, including significant insertions and deletions between the clades. Specifically, ICRSV-A shows insertions at positions 102–104, 114–117, 238–240, and 277–279, while ICRSV-B features insertions at positions 141–144 and 190–198 (Table 4). These sequence differences are consistent across variants within each group, reinforcing the idea that these indels contribute to functional divergence between ICRSV-A and ICRSV-B.

**Table 4:** Summary of Insertions and Deletions Between ICRSV-A and ICRSV-B Based on Coat Protein Gene Alignment.

Position	Type of change	Sequence in ICRSV-A	Sequence in ICRSV-B	Notes
102–104	Insertion	TCC	-	Insertion in all ICRSV-A sequences
114–117	Insertion	CGAG (CAAG in KPI)	**-**	Insertion in all except KPI which has CAAG
141–144	Deletion	**-**	CTCT (15 seqs), CTCA (10 seqs), CTTT (1 seq)	Deletion in ICRSV-A, variable sequences in ICRSV-B
190–198	Deletion	**-**	ACCTTCGGG (24 seqs), ACCCTCGGG (2 seqs)	Deletion in ICRSV-A, variable sequences in ICRSV-B
238–240	Insertion	GAG	-	Insertion in all ICRSV-A sequences
277–279	Insertion	GGC	-	Insertion in all ICRSV-A sequences

In addition to the sequence-based analysis, we accurately predicted the 3D structure of the coat protein for both ICRSV-A and ICRSV-B using AlphaFold2 (Jumper et al., 2021) (Figure 3C). Structural comparisons revealed a root mean square deviation (RMSD) of 1.504 Å, highlighting the structural similarity between the two variants. Key proline (P) insertions were observed, with ICRSV-A showing an insertion at position 35 and ICRSV-B at position 66. Detailed structural visualization using Python Molecular Viewer (PyMOL) confirmed that these insertions occur within a loop region between the helices, which could influence the flexibility and structural conformation of the coat protein. The observed indels, particularly the proline insertions, likely play a role in altering the structural dynamics between the two species, potentially affecting their interaction with host proteins and modes of transmission.

The insertions and deletions observed in the alignment and structural analysis likely reflect differences in the structural or functional properties of the coat protein between the two species, potentially impacting virus-host interactions and transmission dynamics. Given these consistent genetic and structural differences, we propose the formal classification of ICRSV-A and ICRSV-B as two novel species of Indian citrus ringspot virus, based on both phylogenetic and structural criteria. Further structural and functional studies will elucidate the biological significance of these differences and their impact on disease progression in citrus hosts. This classification lays the groundwork for improved diagnostic methods that can differentiate between the two species, which will be crucial for managing the spread of the virus and mitigating its impact on citrus cultivation in affected regions.

### Phylogenetic analysis and clade classification of Citrus yellow vein clearing virus (CYVCV) variants: insights into geographical spread and evolutionary lineages

A maximum likelihood (ML) phylogenetic analysis was performed to elucidate the evolutionary relationships among Citrus yellow vein clearing virus (CYVCV) variants. This analysis focused on two genomic regions: the partial RNA-dependent RNA polymerase (RdRp) region, approximately 280 amino acids in length, and the full-length coat protein (CP) gene, which is about 325 amino acids long. All available sequences from the NCBI GenBank database as of September 2024 were included, encompassing both whole genomes and partial sequences (Supplementary_Excel_Sheet_1.xlsx). To maximize phylogenetic information, the two genomic regions (partial RdRp and full-length CP) were extracted at both the cDNA and protein levels and concatenated for analysis. Despite the high level of amino acid identity among CYVCV variants, even across distantly related strains, the phylogeny was reconstructed using cDNA sequences. This approach allowed for the inclusion of both synonymous mutations (which do not alter the amino acid sequence) and non-synonymous mutations (which result in amino acid changes), thereby increasing the branch lengths in the tree and improving resolution due to the accumulation of a greater number of mutations. The novel CYVCV ML tree generated from the concatenated cDNA sequences is presented in Figure 2. In contrast, the corresponding protein-based tree is provided in Supplementary Figure 1. Despite the lower resolution in the protein tree, both analyses show remarkable concordance in clustering. Based on the ML phylogenetic clustering in both concatenated cDNA and protein trees, we classify the clusters under the following categories:

*Indo-Pak Clade*: In the reconstructed cDNA tree, six CYVCV variants from this analysis were included: P-K-MUK-33 and P-K-MUK-35, sourced from Kinnow plants in Muktsar, and P-K-ABH-19, from Kinnow plants in Abohar, Punjab, India. Both locations are situated in the southwestern part of Punjab, near the Pakistan border. These three variants, along with other sequences from the NCBI database—PALI (from orange) and LKI (from Kinnow) in Ludhiana, Kin-Del (from Kinnow) and DLI (from lemon) in Delhi, ALI (from lemon) in Ahmedabad, AKI (from Kinnow) and ECAI (from Etrog citron) in Abohar, SMI (from orange) in Shri Ganganagar, and PK (from galgal lemon) in Pakistan—form the Indo-Pak Clade. Despite utilizing NNI and SPR methods to optimize nearest neighbor relationships, the statistical support for this branch remains modest. This is partly due to limited sequence coverage, as the three Indian variants only cover the RdRp region and lack the complete coat protein (CP) region, where most of the variation is observed in other variants. Nonetheless, these variants consistently cluster together, forming a distinct group. We have named this cluster the Indo-Pak Clade to distinguish it from other clades in the phylogeny.

*Northern Silk Clade*: The three CYVCV variants analyzed in this study—H-K-SIR-44, H-K-SIR-45, and H-K-SIR-47—were isolated from Kinnow plants in Sirsa, Haryana, and form a well-supported cluster with several additional variants deposited in the NCBI GenBank. These include six variants from lemon, three from Clementine, two from Persian lime, and one from sour orange, all collected in Iran, along with two variants from lemon sourced in Turkey. Due to the geographical distribution of these variants along the ancient Northern Silk Road, historically linking Asia and Europe, we have named this group the Northern Silk Clade to distinguish it from other clades. While analyzing both the cDNA and protein trees, we did observe some switching of the Indian strains between clades, which is likely due to insufficient resolution and missing sequence data for the coat protein region.

**Figure 4:**
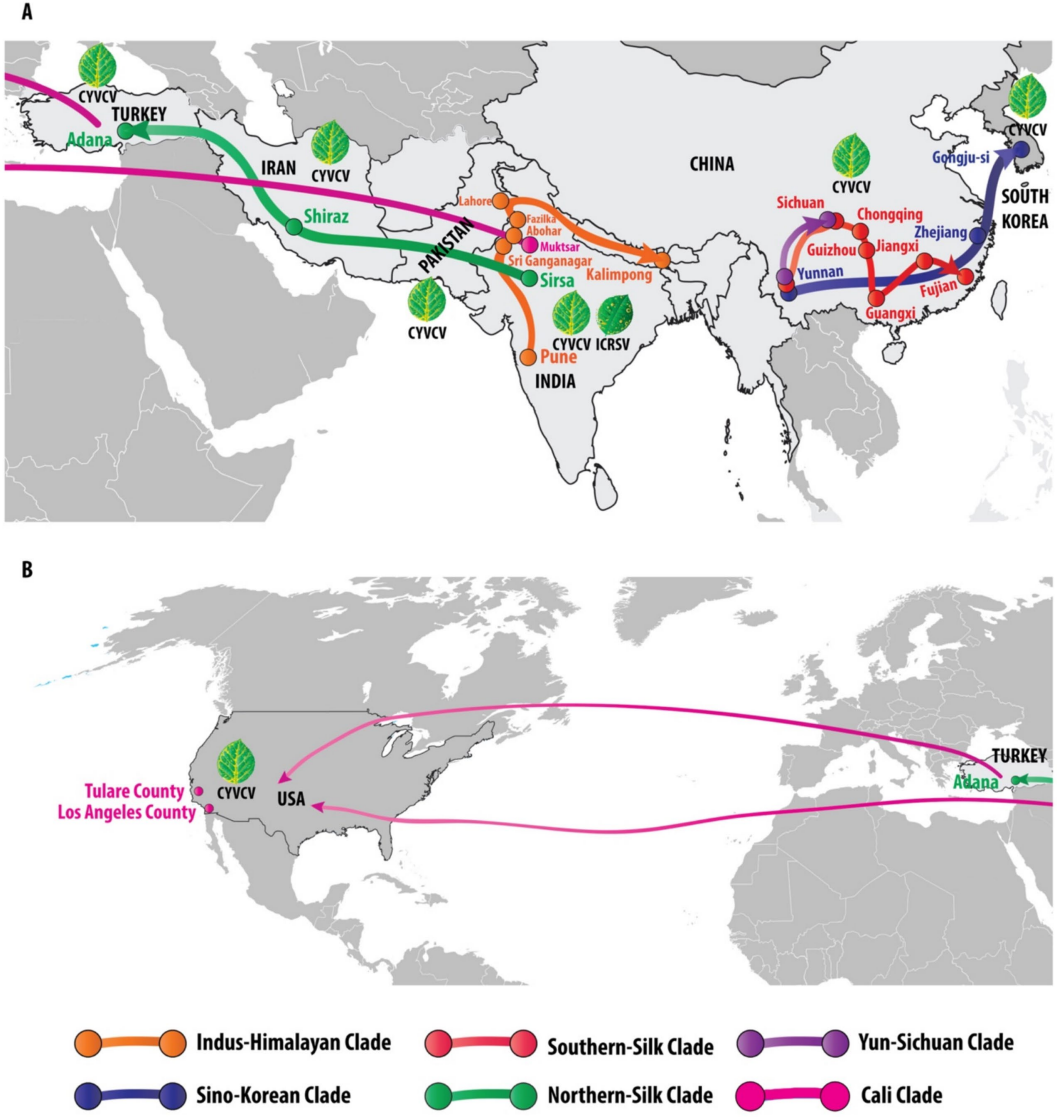
Global phylogeography and transmission pathways of Citrus yellow vein clearing virus (CYVCV): unveiling eight distinct clades across regions. This figure illustrates the global distribution and phylogenetic relationships of Citrus yellow vein clearing virus (CYVCV) across eight distinct clades, identified through phylogenetic analysis. Each clade is represented by a unique color, tracing the virus’s spread across different regions. The Indus-Himalayan Clade (orange) maps the virus’s progression from Pune, Abohar, Fazilka, and Kalimpong in India to Lahore, Pakistan. The Sino-Korean Clade (blue) tracks the virus’s movement from Yunnan and Zhejiang in China to Gongju-si, South Korea. The Northern-Silk Clade (green) follows the virus’s path from Sirsa, India, through Shiraz, Iran, to Adana, Turkey. The Southern-Silk Clade (red) links Yunnan, Sichuan, Guizhou, Guangxi, Jiangxi, and Fujian in China. The Yun-Sichuan Clade (purple) connects Yunnan to Sichuan. The Cali Clade (pink) depicts the movement of the virus either directly from India to the United States or through Turkey, with documented cases in Tulare County and Los Angeles County, California. The figure is divided into two panels: Panel **A** illustrates the virus spread from South Korea to Turkey, while Panel **B** traces the movement from Turkey to the United States. These geographic routes are supported by the phylogenetic analysis, depicted in the color-coded phylogenetic tree in Figure 3. Additionally, illustrations of diseased citrus leaves highlight the characteristic symptoms of CYVCV and ICRSV infections, predominantly reported in India, while other countries (Pakistan, China, South Korea, Iran, Turkey, and the USA) report the presence of CYVCV alone.

*Cali1 Clade*: This clade represents a small but distinct group with strong statistical support, consisting of four variants from California: CA-14, collected from an orange, and CA-15, CA-17, and CA-18, all sourced from lemons in California, USA. The Cali1 Clade is positioned between the Indo-Pak Clade and the Northern Silk Clade in Figure 4, presenting two possible scenarios for the evolutionary history of these variants. The first scenario suggests that these Californian CYVCV variants may have traveled along the Silk Road to Europe, eventually reaching Spain. From there, citrus varieties, including oranges, were introduced to Florida by Spanish explorers such as Juan Ponce de León and later spread to California. The second scenario posits a more direct introduction of the virus into California from regions like India, Pakistan, Iran, or Turkey, with India being a particularly strong possibility. Given the distribution of CYVCV variants along the Northern Silk Route, the first scenario appears more plausible. This distinct Californian cluster, named Cali1 to differentiate it from others, highlights its unique geographical association with the state of California.

*Cali2 Clade*: This clade comprises 13 Californian variants that form a well-defined cluster adjacent to the Northern Silk Clade, with whole genome sequences available in the NCBI database (Sun and Yokomi, 2024). These variants were collected from a wide range of citrus species, including mandarin, citron, Bearss lime, lemon, Mexican lime, grapefruit, kumquat, Eureka lemon, and Kaffir lime, demonstrating that CYVCV has successfully spread across multiple citrus hosts in California. The initial branch length of this clade is notably longer, indicating a phase of mutational accumulation, which may have been driven by adaptation to new hosts or specific environmental conditions in California. Over time, the subsequent stabilization of mutation rates across the variants suggests that the virus has adapted to local conditions, reflecting its evolutionary response to its new environment. We have named this group the Cali2 Clade to distinguish it from other clades and to highlight its unique evolutionary trajectory within California.

*Indus-Himalayan Clade*: Adjacent to the Cali2 Clade, this new clade comprises variants from multiple regions, including Fazilka (collected from malta and kinnow), Abohar (from Etrog citron), Sri Ganganagar (from malta), and Pune (from kinnow). These variants form a strong cluster alongside a variant collected from lemon in Kalimpong. A CYVCV variant from Eureka lemon in Pakistan is positioned between the Cali2 Clade and this new clade. The unique geographic distribution of these variants spans both northwestern and northeastern India, as well as Pakistan, covering a wide area that reflects the diverse agricultural landscapes and climates of the Indus River Valley and the Himalayan foothills. This broad distribution suggests potential historical or trade routes that may have facilitated the spread of the virus across these regions. We have named this group the Indus-Himalayan Clade to reflect its vast geographic range and to distinguish it from other clades. This clade consistently appears in both the cDNA and protein trees, where it forms a neighboring clade to the Cali2 Clade in both phylogenies.

*Yun-Sichuan Clade*: Adjacent to the Indus-Himalayan Clade, the Yun-Sichuan Clade is a well-supported group that includes variants from Eureka lemon in Yunnan, five variants from Eureka lemon in Sichuan, one from lemon in Sichuan, and another from an unrecognized location in China. In the protein tree, this clade also includes variants from sweet orange and Satsuma in Yunnan. The cDNA tree, offering better phylogenetic resolution by incorporating both synonymous and non-synonymous mutations, provides clearer insights into the evolutionary relationships within this clade. The stable clustering in the cDNA tree suggests long-term evolutionary divergence. Geographically, Yunnan’s proximity to northeastern India and Myanmar, aligning with the reported origin of citrus (Wu et al., 2018), suggests a possible route for CYVCV dispersal via agricultural trade or natural ecological spread, positioning this region as a potential hub for virus transmission.

*Sino-Korean Clade*: Adjacent to the Yun-Sichuan Clade is the Sino-Korean Clade, which comprises three variants from Yunnan, collected from orange, *Citrus reticulata*, and Qingpiju, along with four variants from Zhejiang, including two from *C. reticulata × C. sinensis* hybrids and two from *Citrus medica*. In the protein tree, this clade also includes two variants from Sichuan, collected from lemon and sweet orange. These Chinese variants cluster with four variants from South Korea: two collected from Persian lime, one from *Citrus limon*, and one from calamansi. Due to the characteristic eastward spread across the Yellow Sea between China and Korea, we have named this group the Sino-Korean Clade. This geographic distribution suggests a possible route for viral dispersal across East Asia, potentially facilitated by agricultural practices or trade connections between China and South Korea. The clustering of variants from different citrus species may also indicate a broader host range adaptability of CYVCV in this region.

*Southern-Silk Clade*: This is the largest CYVCV clade, comprising seven variants from Yunnan (lemon, satsuma, sweet orange, asumi, nichinan 1 wase), two from Sichuan (lemon, sweet orange), two from Guanxi (tangerine, pummelo), three from Chongqing (zaoyangxiaoyezhi, Tarocco, Eureka lemon), three from Guangdong (Shatangju, pummelo, Jiaogan), one from Guizhou (pummelo), two from Hunan (pummelo, tangerine), one from Fujian (Pokan), and two from Jiangxi (tangerine, sweet orange). We named this group the Southern-Silk Clade because the geographic regions represented by the variants—primarily in southern China, including Yunnan, Sichuan, and Guangdong—were historically part of the ancient Southern Silk Road trade network. This ancient trade route may have played a significant role in the spread of citrus fruits and CYVCV variants across these regions.

### Geographical distribution and phylogenetic relationships of Citrus yellow vein clearing virus (CYVCV)

The analysis of Citrus yellow vein clearing virus (CYVCV) revealed eight distinct clades based on geographic distribution and phylogenetic relationships. The Indus-Himalayan Clade demonstrated the virus’s spread across northern India, including Pune, Abohar, Fazilka, and Kalimpong, and extended to Lahore, Pakistan, confirming a strong presence in the north-western region of India. The Sino-Korean Clade traced virus movement across Yunnan and Zhejiang provinces in China to Gongju-si, South Korea, supporting the hypothesis of eastward transmission within East Asia. The Northern-Silk Clade followed a westward path from Sirsa, India, through Iran (Shiraz) to Adana, Turkey, aligning with historical trade routes that may have facilitated the virus’s spread. The Southern-Silk Clade demonstrated a strong clustering in southern China, covering regions such as Yunnan, Sichuan, Guizhou, Guangxi, Jiangxi, and Fujian. The Yun-Sichuan Clade revealed a direct connection between Yunnan and Sichuan, reinforcing regional transmission patterns. Finally, the Cali Clade illustrated two probable transmission routes to California, USA: one directly from India and another through Turkey, with CYVCV detected in both Tulare and Los Angeles Counties. Notably, the presence of CYVCV was reported across Pakistan, China, South Korea, Iran, Turkey, and the USA, while co-infection with ICRSV was unique to the north-western region of India. The phylogenetic analysis, supported by color-coded maps (Figure 4) and illustrations of symptomatic citrus leaves, reinforces these geographic transmission patterns, indicating a distinct spread across key agricultural and trade regions.

## Discussion

The identification of two novel species of Indian citrus ringspot virus (ICRSV-A and ICRSV-B), alongside the development of a sensitive RT-PCR assay, marks a significant advance in citrus virology. This study sheds light on the evolutionary dynamics of these mandariviruses and provides practical diagnostic tools to mitigate their growing threat, especially in regions where both viruses coexist. Notably, the phylogenetic classification of ICRSV into two distinct species represents the first clear genetic delineation within this virus, highlighting significant sequence divergence in the coat protein gene with potential implications for transmission dynamics and virulence. This finding lays a foundation for future epidemiological studies aimed at understanding how these species may interact with diverse citrus hosts.

The RT-PCR assay developed in this study targets conserved regions of the coat protein gene, allowing for the simultaneous detection of ICRSV and CYVCV in a single reaction. Offering enhanced specificity and sensitivity over traditional methods such as ELISA and Immunosorbent Electron Microscopy (ISEM), the assay addresses key limitations like cross-reactivity and reduced sensitivity in mixed infections (Alshami and Ahlawat, 2003). Moreover, it is a cost-effective alternative to resource-intensive methods like RPA-based diagnostics and RT-qPCR, making it highly practical for large-scale use in resource-limited settings. The ability to detect both viruses simultaneously not only reduces diagnostic costs but also provides a more streamlined approach to monitoring outbreaks in endemic regions. This capability is critical for managing mandarivirus outbreaks, particularly in regions where co-infections exacerbate symptoms and lead to greater economic losses (Meena and Baranwal, 2016).

Structural modeling of the ICRSV coat protein gene identified unique proline insertions in ICRSV-A, which may significantly impact its interactions with host plants and insect vectors. These proline residues are known to enhance protein flexibility, potentially improving viral assembly, transmission efficiency, and immune evasion. Comparable effects have been documented in other aphid-transmitted viruses, underscoring the importance of further investigating these structural differences through viral fitness assays and transmission studies (Bhardwaj and Purohit, 2020). Elucidating the functional role of these insertions could offer insights into how structural variation contributes to virulence and host adaptability.

The coat protein cDNA sequence analysis of CYVCV revealed valuable insights into sequence conservation and geographic trends. Pairwise sequence identity analysis showed intra-country similarities exceeding 98% in regions such as India, Pakistan, Iran, and Turkey, supporting phylogenetic evidence that positions the Indian subcontinent, including India and Pakistan, as a potential epicenter for CYVCV spread. Two major transmission routes were identified: one extending westward through Iran and Turkey to the USA, and another moving eastward through China to South Korea. Hierarchical clustering (Figure 2C) and similarity heatmaps (Figure 2B) further reinforced these trends, highlighting strong clustering among neighboring regions and slightly lower conservation (~97%) in isolates from more distant locations like the USA and South Korea. These findings underscore the significant role of geographic and trade dynamics in shaping CYVCV transmission and evolution. The identification of these transmission routes emphasizes the importance of monitoring international citrus trade to limit further viral dissemination.

CYVCV poses a significant threat to diverse citrus species and cultivars, including *Citrus limon*, *Citrus aurantium*, *Citrus siensis*, and *Citrus reticulata*. Symptoms such as yellow vein clearing, vein water-soaking, and chlorosis are particularly severe in susceptible cultivars like Etrog citron. Infected fruits, especially those of ‘Satsuma mandarin’ and ‘Eureka’ lemon, frequently exhibit malformations, such as groove-like depressions (Li et al., 2017). Notably, symptom severity is influenced by environmental conditions, with higher temperatures reducing symptoms or resulting in asymptomatic infections (Chen et al., 2014). These observations highlight the need to consider environmental factors when designing effective management strategies. Developing region-specific management protocols that account for these environmental influences could significantly improve disease control efforts.

Diagnostic differences between CYVCV and ICRSV are also noteworthy. CYVCV is associated with yellow vein clearing and fruit malformations, whereas ICRSV is characterized by distinct ringspot lesions and chlorotic patterns on leaves and fruits (Prabha and Baranwal, 2011). The RT-PCR assay developed in this study provides a reliable tool for distinguishing between these viruses, particularly in regions with mixed infections, where traditional methods often fall short.

Significant research in China has advanced our understanding of CYVCV, focusing on its molecular characteristics, transmission mechanisms, and prevention strategies (Liu et al., 2020; Zhou et al., 2017). Robust measures, such as early virus detection and propagation of virus-free nursery trees, have proven critical in mitigating its impact on citrus production (Chen et al., 2014). The inclusion of CYVCV on the EPPO Alert List underscores its potential threat to Mediterranean citrus production, as evidenced by recent detections in Campania, Italy (EPPO, 2024). These findings serve as a reminder of the global nature of CYVCV’s threat, calling for coordinated international research and policy interventions.

The global spread of CYVCV, as evidenced by our phylogenetic analysis, reflects the role of trade and agricultural practices in its dissemination. The identification of distinct clades, such as the Indus-Himalayan and Cali Clades, highlights historical and contemporary movement of citrus planting material. These findings emphasize the importance of stringent quarantine measures and regulated trade to limit further spread. The detection of CYVCV in California, a vital citrus-producing region, is particularly concerning and underscores the importance of controlling infected plant material to prevent further economic losses. As trade networks expand, integrating diagnostic tools into global certification programs could play a pivotal role in mitigating disease spread.

In conclusion, this study presents significant advancements in the detection and understanding of mandariviruses, particularly ICRSV and CYVCV. The development of a novel RT-PCR assay, capable of detecting both viruses in single and mixed infections, combined with the discovery of two new ICRSV species, provides valuable tools for citrus disease management. As global trade and environmental changes continue to influence the spread of plant pathogens, the insights provided by this study are essential for protecting citrus production worldwide and ensuring the sustainability of this economically important industry. Future research should prioritize understanding the ecological and functional impacts of genetic variability in these viruses, especially their interactions with diverse citrus hosts and vector populations.

## Conclusion

This study provides critical advancements in the detection and phylogenetic understanding of two major viral pathogens affecting citrus crops: Indian citrus ringspot virus (ICRSV) and Citrus yellow vein clearing virus (CYVCV). By developing and validating novel RT-PCR primers targeting conserved regions of the coat protein (CP) and RNA-dependent RNA polymerase (RdRp) genes, we have established a highly sensitive and specific assay for the simultaneous detection of these mandariviruses, representing a significant innovation over existing diagnostic methods for mixed infections. This assay offers significant practical applications for early diagnosis and disease management.

Our comprehensive phylogenetic analysis revealed the presence of two novel ICRSV species, ICRSV-A and ICRSV-B, which were classified based on significant genetic divergence, unique insertions and deletions (indels), and structural differences in the coat protein. This genetic differentiation is the first to formally establish species-level divergence within ICRSV, paving the way for improved diagnostics and tailored management approaches. Furthermore, the identification of eight distinct CYVCV clades across diverse geographic regions highlights the virus’s widespread presence and genetic variability, reinforcing the need for vigilant monitoring and containment efforts.

These findings not only expand our understanding of the genetic diversity and evolution of CYVCV and ICRSV but also provide essential tools for their accurate identification. The novel RT-PCR assay we developed has the potential to significantly improve virus detection and co-infection diagnosis, which is critical in regions where both viruses coexist and pose a threat to citrus production. Its integration into global citrus certification and quarantine programs is a crucial step in limiting transboundary movement of these pathogens, ensuring sustainable citrus production and reducing economic losses worldwide.

In conclusion, this study bridges important knowledge gaps in citrus virology, offering both molecular and practical solutions for managing viral threats to one of the world’s most valuable fruit crops. Future research should focus on elucidating the biological and epidemiological impacts of the genetic and structural differences identified between ICRSV-A and ICRSV-B, particularly their roles in host adaptation and vector interactions. These insights will be critical for refining disease management strategies and safeguarding global citrus production in the face of emerging viral threats.

## Materials and methods

### Sample collection and processing

Eleven confirmed positive seedlings infected with Indian citrus ringspot virus (ICRSV) and Citrus yellow vein clearing virus (CYVCV) were maintained within an insect-proof screen house at the Indian Council of Agricultural Research - Central Citrus Research Institute (ICAR-CCRI), Nagpur, Maharashtra, India. These seedlings served as the primary source for validating novel primers using RT-PCR. Additionally, samples previously analyzed by Kokane et al. (2020a) were included to enhance the robustness of the validation study. Symptomatic portions of mature leaves, displaying prominent chlorotic rings, were carefully excised, immediately frozen in liquid nitrogen, and ground into a fine powder. The powdered samples were stored at −80°C to maintain sample integrity for subsequent molecular analysis.

### Designing of RT-PCR primers

To design the primers, reference sequences of the coat protein (CP) gene were retrieved from the NCBI database.^1^ The sequences were aligned using ClustalW in MEGA 7 software, identifying conserved regions within the CP and RNA-dependent RNA polymerase (RdRp) genes. Primer3 version 4.1.0^2^ was used to design novel primers targeting these conserved regions. The primers were synthesized by Integrated DNA Technologies (IDT), Bangalore, India (Table 1), and validated through RT-PCR analysis.

### Genomic RNA isolation

Total genomic RNA was extracted from 100 mg of ground citrus tissue, including both healthy and infected samples, using the RNeasy Plant Mini Kit (Qiagen, Germany). The RNA extraction followed the manufacturer’s guidelines, ensuring high-quality and contaminant-free RNA. The isolated RNA was dissolved in 50 μL of TE (Tris-EDTA) buffer and stored at −80°C for later use. RNA quality and quantity were measured using a NanoDrop 2000 Spectrophotometer (Thermo Scientific), ensuring it was suitable for subsequent analysis.

### Detection of mandariviruses using RT-PCR

RT-PCR was performed using total RNA extracted from each sample, applying novel forward (Mand-D-CP1-F) and reverse (Mand-D-CP1-R) primers specifically targeting the coat protein gene. The RT-PCR process followed a two-step protocol adapted from Kokane et al. (2020a), beginning with cDNA synthesis. cDNA synthesis was carried out in a 15 μL reaction containing 1X first-strand buffer, 0.5 mM dNTPs, 15.6 U RNasin, 0.4 μM reverse primer (Mand-D-CP1-F/CP1-R for the coat protein), 6 μL of total RNA, and 120 U of M-MLV reverse transcriptase. The reaction proceeded at 42°C for 50 min for reverse transcription, followed by a 10-min extension at 72°C.

For PCR amplification, 1.7 μL of the synthesized cDNA was used in a 25 μL reaction volume containing 1X PCR buffer, 0.2 μM of each primer (Mand-D-CP1-F/CP1-R), 0.2 mM dNTPs, 1.5 mM MgCl2, and 1.25 U of DNA polymerase (Promega, Madison). The PCR was carried out using a Bio-Rad 100TM Thermal Cycler with the following cycling conditions: an initial denaturation step at 94°C for 3 min, followed by 35 cycles of 94°C for 30 s (denaturation), 55°C for 45 s (annealing), and 72°C for 50 s (extension), with a final extension at 72°C for 10 min. Amplified PCR products were visualized using 1.2% agarose gel electrophoresis.

Further validation was performed using specific primers for ICRSV (ICRSV-CP-3F/3R) and CYVCV (RdRp gene-specific primers 391fw/121rev) at an annealing temperature of 58°C.

### Validation of novel primer detection assay

To validate the novel primers, 49 field samples suspected of infection with ICRSV and CYVCV were analyzed. The assay included RNA from samples infected with other major citrus pathogens such as Citrus tristeza virus (CTV), Citrus yellow mosaic virus (CYMV), *Candidatus* Liberibacter asiaticus (the causative agent of Huanglongbing), and citrus phytoplasma. RNA/DNA from healthy citrus plants maintained in the same screen house served as controls. The RT-PCR assays were performed in triplicate to ensure reproducibility and the reliability of the results.

### RT-PCR products sequencing and analysis

Amplified RT-PCR products of ICRSV and CYVCV were purified from agarose gels using the GenElute Gel Purification Kit (Sigma-Aldrich, India). The purified products were sequenced using the Sanger sequencing method at Eurofins Genomics, Bengaluru, India. Sequence data were analyzed using BioEdit Software version 7.2, and chromatograms from forward and reverse sequences were inspected for quality. Sequence alignments were performed, and BLAST searches were conducted to confirm sequence identity. Assembled gene sequences were submitted to GenBank via the BankIt submission tool^3^.

### Pairwise sequence identity analysis and Heatmap construction

Nucleotide and protein sequences of CYVCV and ICRSV variants were aligned using the Muscle algorithm to ensure accurate assessments of genetic variability. Pairwise sequence identity values were calculated for both intra-and inter-country comparisons, focusing primarily on the coat protein (CP) cDNA region. Heatmaps were generated using the seaborn library in Python to visually represent sequence identity values, which ranged from 95 to 100%. Darker shades in the heatmap indicated higher sequence identity, while lighter shades represented lower similarity. Intra-country sequence similarities were averaged to highlight local conservation patterns, while inter-country comparisons were analyzed to uncover broader geographic trends. These analyses provided insights into the genetic clustering of CYVCV variants, revealing key transmission routes and corroborating findings from phylogenetic analyses.

### Sequence retrieval, extraction of genomic location, sequence alignment, and phylogeny reconstruction

Depending on the amino acid or nucleotide sequences, BLASTp, TBLASTn, or BLASTn searches were performed using either the RNA-dependent RNA polymerase (RdRp) or the coat protein (CP) as queries against all GenBank-deposited sequences, including full-genome sequences available at NCBI. Both partial and full-genome sequences of ICRSV and CYVCV were retrieved and downloaded for analysis (Supplementary_Excel_Sheet_1.xlsx).

Local standalone BLAST searches were performed against the retrieved genomic sequences. In local searches, the amino acid sequences of RdRp or CP were used as a query in TBLASTn searches to identify corresponding genomic regions. These nucleotide sequences were then translated into three frames using the SMS Translator tool^4^, and the relevant genomic regions were manually extracted.

A novel phylogenetic reconstruction approach was employed using concatenated nucleotide and protein sequences of the RdRp and CP regions. This method was guided by our prior experience with an unrelated virus, where it yielded highly reliable results (Ghosh et al., 2022). Phylogeny was performed using DNA sequences to capture both synonymous and non-synonymous mutations. Sequence alignment was carried out using MUSCLE (Edgar, 2004), and maximum likelihood (ML) trees were generated using PhyML v3.0 (Guindon et al., 2010; Guindon and Gascuel, 2003). The best-fit evolutionary models were identified using the Akaike Information Criterion (AIC), with the General Time Reversible (GTR) substitution model used for nucleotide sequences. Tree topology, branch lengths, equilibrium frequencies, and substitution rates were estimated. Clade support was assessed using the SH-like approximate likelihood ratio test (Anisimova et al., 2011). Phylogenetic trees were visualized and edited using iTol version 2.0 (Letunic and Bork, 2019), and high-resolution images were generated using Adobe Illustrator CS6 for publication.

### AlphaFold2-based protein structure prediction

To gain structural insights into Indian citrus ringspot virus (ICRSV), we utilized AlphaFold2 (Jumper et al., 2021) to predict the 3D structures of the coat proteins (CP) from both ICRSV-A and ICRSV-B. The amino acid sequences of these two distinct variants were used as input for AlphaFold2, generating high-confidence structural models. The resulting models were visualized and analyzed using Python Molecular Graphics (PyMOL), with particular emphasis on identifying potential structural differences, such as insertions and deletions between ICRSV-A and ICRSV-B. Root Mean Square Deviation (RMSD) values were calculated to quantify the structural deviations between the predicted models of ICRSV-A and ICRSV-B. These analyses provide deeper insights into the structural biology of ICRSV variants, revealing potential differences in folding and stability that may influence their behavior in the host environment.

## Data Availability

The datasets presented in this study can be found in online repositories. The names of the repository/repositories and accession number(s) can be found in the article/Supplementary material.

## Footnotes

^1^ http://www.ncbi.nlm.nih.gov/

^2^ http://primer3.ut.ee

^3^ https://www.ncbi.nlm.nih.gov/WebSub/

^4^ https://www.bioinformatics.org/sms2/trans_map.html
