# Supplementary material for: Development of diagnostic tools and discovery of two novel Indian citrus ringspot virus species: insights into global mandarivirus phylogeography
Source: Front Microbiol. 2025 Feb 14;16:1513291. doi: 10.3389/fmicb.2025.1513291 (PMC11868068; doi:10.3389/fmicb.2025.1513291)

**Supplementary Figure 1: Protein-Based Maximum Likelihood (ML) Phylogenetic Tree of Citrus Yellow Vein Clearing Virus (CYVCV) Variants**

This figure presents the ML phylogenetic tree based on the full-length coat protein (CP) sequences of Citrus Yellow Vein Clearing Virus (CYVCV) variants. The tree includes all available CYVCV protein sequences from the NCBI GenBank database as of September 2024. Bootstrap values of 80% and higher are shown at the nodes, supporting the classification of distinct evolutionary lineages. The protein-based tree offers complementary insights to the cDNA-based analysis provided in the main text.


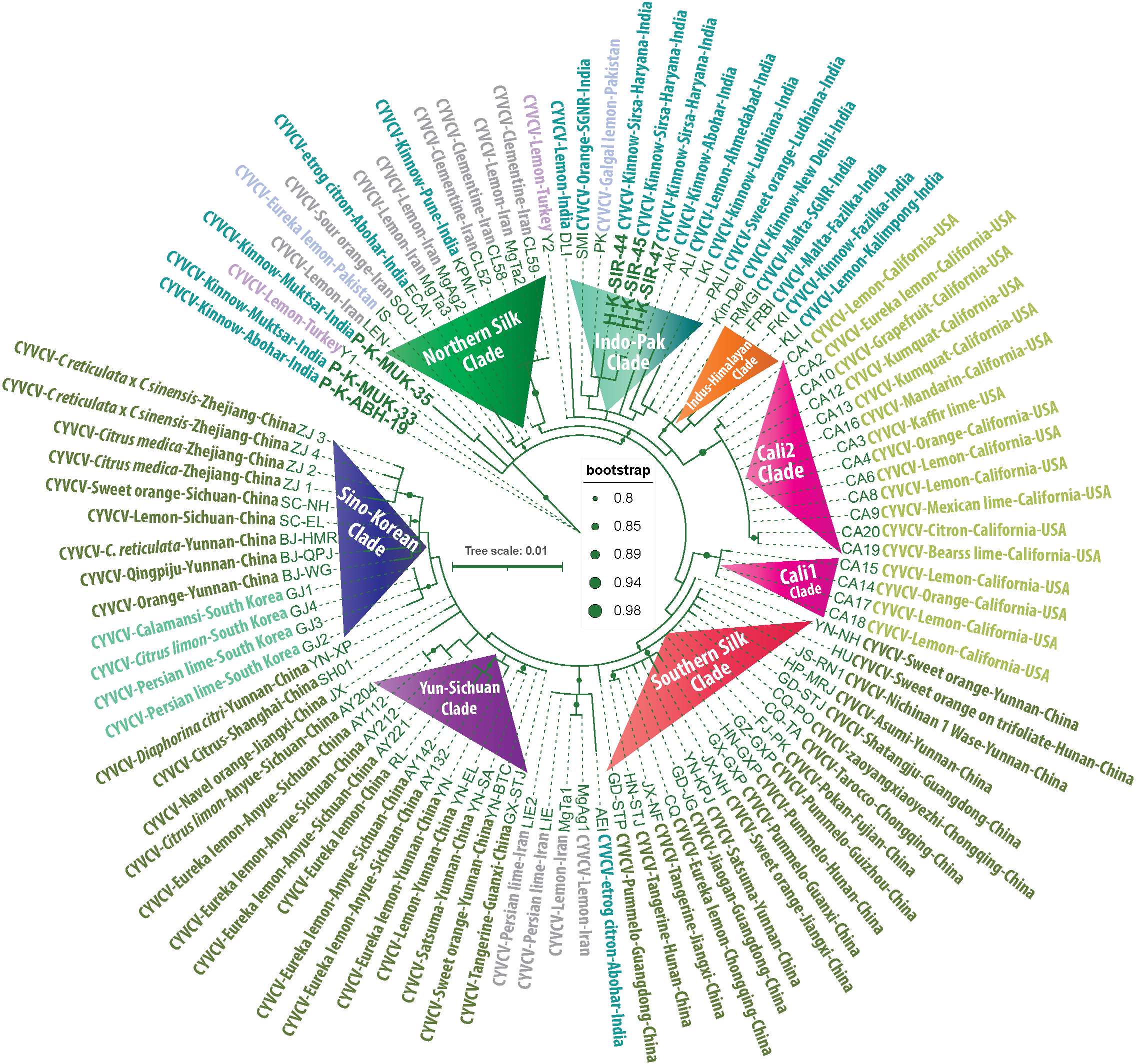


**Supplementary Figure 2: Heatmap of Intra- and Inter-Country Sequence Similarities in the Coat Protein (CP) Region of CYVCV**

This heatmap illustrates the pairwise sequence similarity percentages in the coat protein (CP) region of Citrus Yellow Vein Clearing Virus (CYVCV) isolates across various countries and regions. High intra-country sequence conservation is observed, particularly in India, Pakistan, Iran, and Turkey, with values exceeding 98%. Inter-country similarities demonstrate geographic trends, with slightly lower similarities observed for isolates from distant regions such as China, South Korea, and the USA. The heatmap emphasizes clustering patterns among geographically proximate countries and highlights evolutionary constraints in the coat protein.


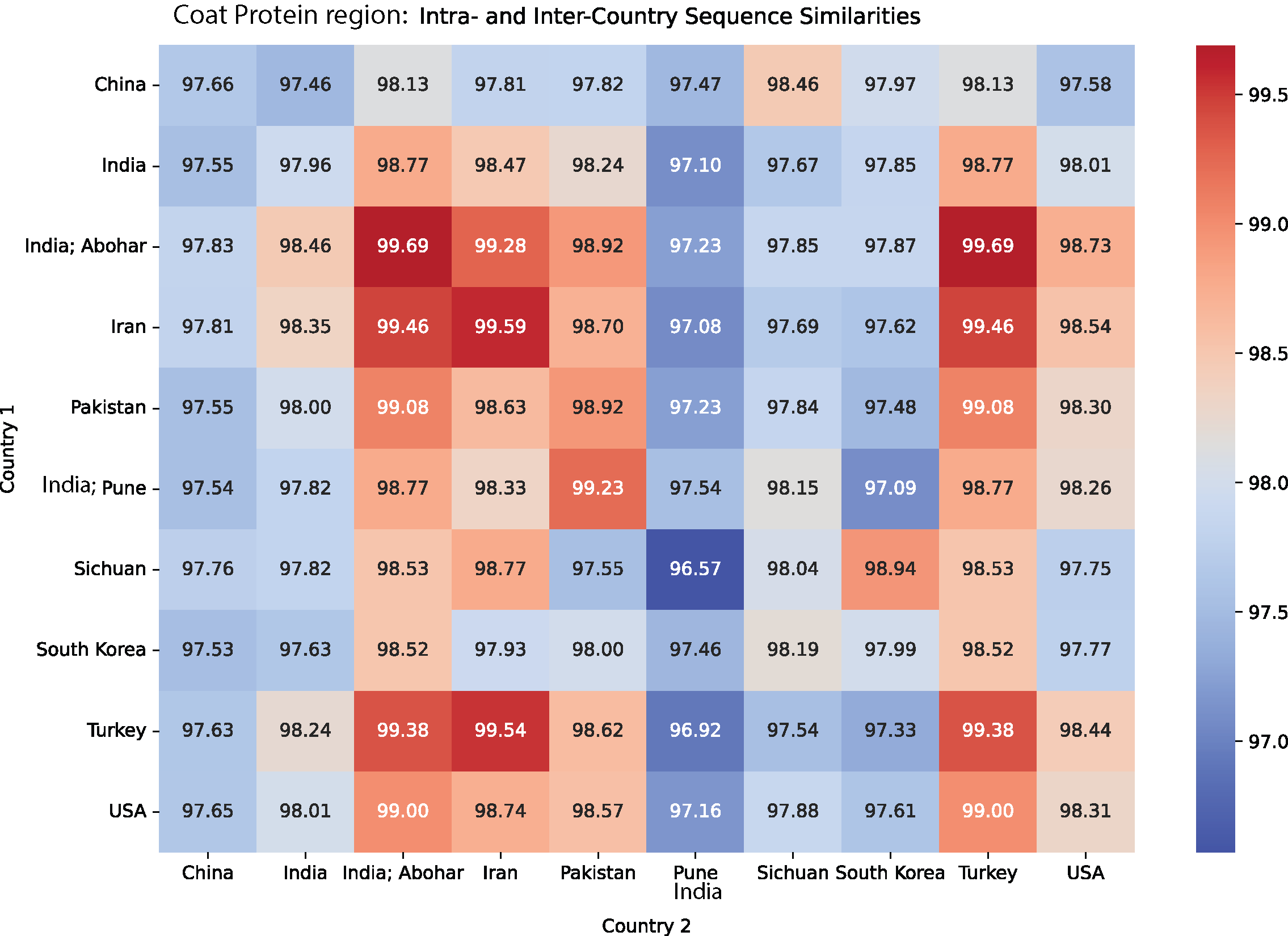


**Supplementary Figure 3: Heatmap of Intra- and Inter-Country Sequence Similarities in the RNA-Dependent RNA Polymerase (RdRP) Region of CYVCV**

This heatmap shows pairwise sequence similarity percentages in the RNA-dependent RNA polymerase (RdRP) region of Citrus Yellow Vein Clearing Virus (CYVCV) isolates across various countries and regions. Both intra- and inter-country sequence conservation patterns are evident, with high similarities (>97%) within neighboring countries such as India, Pakistan, and Iran. Isolates from more distant regions, including the USA, South Korea, and China, exhibit slightly lower sequence conservation. The heatmap provides a comparative visualization of sequence variation in the RdRP region, underscoring its evolutionary dynamics.


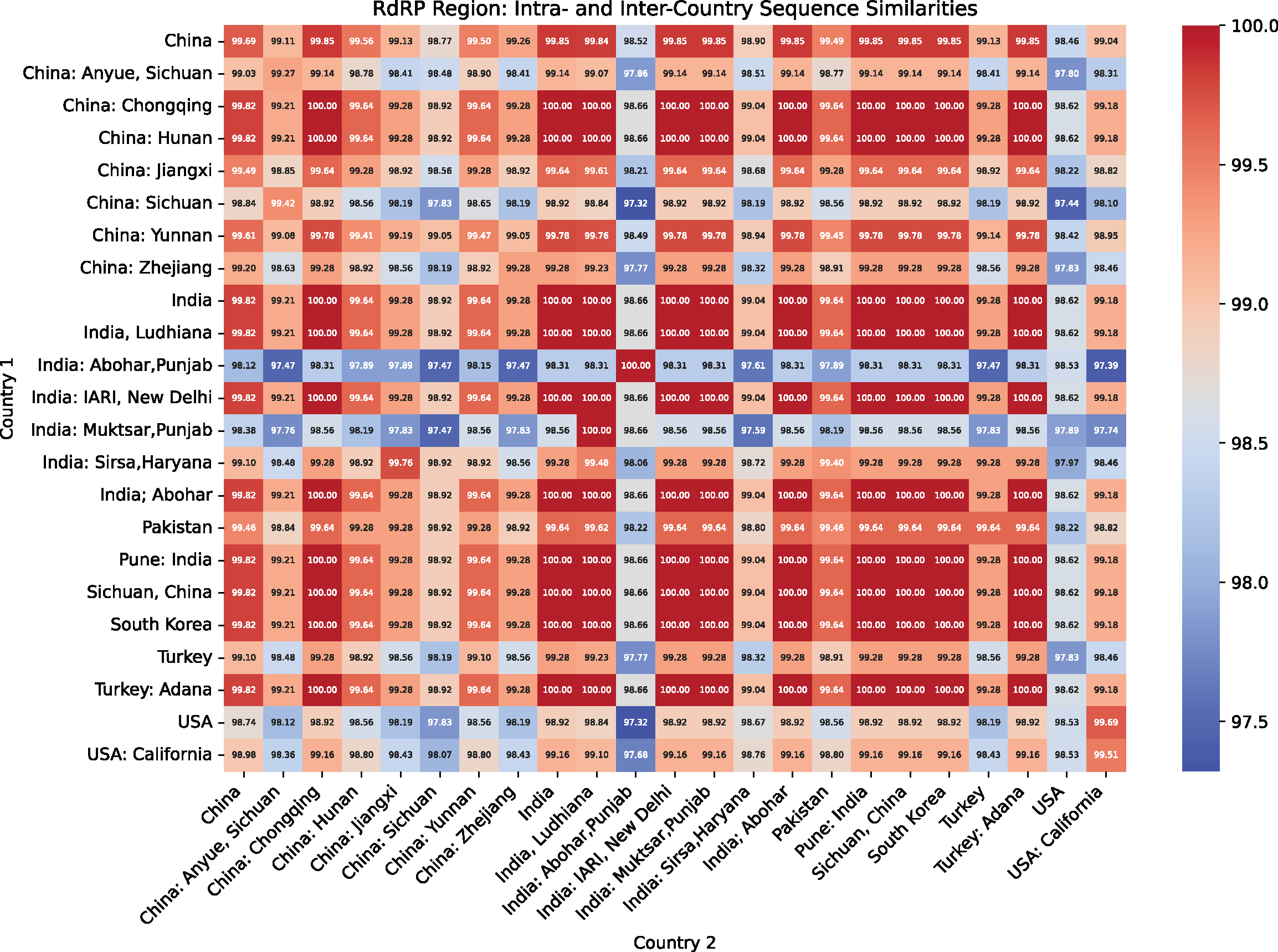

Supplement: Supplementary file 2 [file Data_Sheet_1.docx]
